# Oral and Periodontal Health in Patients with Alzheimer’s Disease and Other Forms of Dementia – A Cross-sectional Pilot Study

**DOI:** 10.3290/j.ohpd.b1248937

**Published:** 2021-04-22

**Authors:** Oliver Laugisch, Andreas Johnen, Walter Buergin, Sigrun Eick, Benjamin Ehmke, Thomas Duning, Anton Sculean

**Affiliations:** a Principal Investigator, Honorary Researcher, Department of Periodontology and Peri-Implant Diseases, Philipps University, Marburg, Germany; former Research Associate, Department of Periodontology and Conservative Dentistry, University Hospital Münster, Münster, Germany. Study concept, design and management, wrote the manuscript.; b Neuropsychologist, Section for Neuropsychology, Department of Neurology, University Hospital Münster, Münster, Germany. Performed neuropsychological diagnostics of all patients, proofread the manuscript.; c Former Statistician, Research Section, School of Dental Medicine, University of Bern, Bern, Switzerland. Performed statistical evaluation of all data, proofread the manuscript.; d Professor, Department of Periodontology, School of Dental Medicine, University of Bern, Bern, Switzerland. Contributed substantially to study conception and design, proofread the manuscript.; e Professor and Chair, Department of Periodontology and Conservative Dentistry, University Hospital Münster, Münster, Germany. Contributed substantially to conception and design of the study, proofread the manuscript; f Professor, Department of Neurology, University Hospital Münster, Münster, Germany. Contributed substantially to conception and design of the study, proofread the manuscript; g Professor and Chair, Department of Periodontology, School of Dental Medicine, University of Bern, Bern, Switzerland. Contributed substantially to study conception, study design, and discussion, proofread the manuscript.

**Keywords:** Alzheimer’s disease, dementia, dental care, oral health, periodontal disease

## Abstract

**Purpose:**

Systemic inflammation is characteristic for the pathogenesis of Alzheimer’s disease (AD) and is responsible for the accumulation of its disease-specific Tau-protein and β-amyloid plaques. Studies focusing on an association with periodontitis showed worse periodontal conditions in patients with dementia, but until now, no study has investigated the differences between AD and other forms of dementia (noAD/DEM). Expecting severe periodontal disease in AD, the aim of this pilot-study was to compare the periodontal and dental status in patients with either AD or noAD/DEM.

**Materials and Methods:**

Twenty patients recently diagnosed with AD and 20 with noAD/DEM between the ages of 50 and 70 years were recruited at the Department of Neurology, University Hospital, Münster, Germany and clinically examined at the Department of Periodontology, School of Dental Medicine, Münster, Germany. Neuropsychological testing, levels of Tau-protein and β-amyloid in serum and liquor were used to distinguish between both groups. Dental and periodontal parameters such as clinical attachment loss (CAL), probing pocket depth (PPD), bleeding-on-probing (BOP), radiographic bone loss, full-mouth plaque score (FMPS), and missing and restored teeth were recorded.

**Results:**

Periodontitis was diagnosed in all patients. Patients with AD presented mean BOP of 54.7 ± 31.1% and radiographic bone loss of 42.5 ± 25.3%; the mean BOP of those with noAD/DEM was 52.0 ± 23.7% and radiographic bone loss was 40.9 ± 32.3%. There was also no statistically significant difference regarding other periodontal and dental parameters.

**Conclusions:**

Both patients with AD and noAD/DEM had periodontal disease. Consequently, patients with all forms of dementia (AD/other) need special dental care to improve periodontal and oral health.

In 1906, Alois Alzheimer described for the first time the “strange disease” of his patient Auguste D., which was later named after him. Around 110 years later, dementia – with Alzheimer’s Disease (AD) being most frequent – is a challenge for health-systems worldwide.^41^ Neuropsychological testing and determination of certain markers in cerebrospinal fluid (CSF), such as total tau-protein and β-amyloid1-42 (Aβ1-42), allows distinguishing between different forms of dementia.^9,15^

Sooner or later, this disease engenders important physical, intellectual and social dependence, which has a severe impact on the social life of the patient and those around him/her. Dementia is the main reason for the high dependency of the elderly and admissions to nursing homes. At present, 40% of AD patients live in these institutions.^42^

Therefore, AD and all other forms of dementia (noAD/DEM) have become a public-health challenge. In 2010, the German government under the patronage of the German Alzheimer’s Association founded the Alzheimer-Alliance to organise and manage comprehensive care for AD patients and caregivers.^5^

According to the modified amyloid hypothesis, in AD, extracellular amyloid-β (Aβ) plaques and intracellular neurofibrillary tau tangles are the pathognomonic hallmarks for the diagnosis and progression of AD.^15^ Lower levels of total tau-protein and elevated Aβ1-42-levels in CSF were recognised as important biomarkers and were identified as therapeutically relevant molecular targets.^9^ Pathological lesions are strongly associated with progressive loss of neurologic capacities, and it is hypothesised that prevention of these accumulations may improve symptoms of this disease.^25^

A systematic inflammation apparently seems to play a significant role in the onset and progression of AD.^11,16,34^ Elevated systemic levels of inflammatory mediators, i.e. interleukin (IL)-1β, IL-6 und tumor necrosis factor α (TNF-α), have been demonstrated to be associated with the neuronal degeneration found in AD.^3,26^ Many studies indicate that an infection triggering an inflammatory response may be linked to AD.^18,20,39^

In this context, periodontitis has gained increasing attention.^13,19,21,36^ It has been suggested that the production of Aβ1-42 plaques and extracellular tau tangles are triggered via a systematic inflammatory reaction.^39^ Although periodontal disease has been documented in all forms of dementia, to date, no study has analysed the differences between AD and noAD/DEM. Therefore, we compared the oral and periodontal health of patients affected by AD and other forms of dementia.

## Materials and Methods

### Experimental Design and Patients

This study was evaluated and approved by the corporate Ethics Committee of the medical association in Westfalen-Lippe, Germany, and the Westfälische-Wilhelms University of Münster, Germany (# 2014-066-f-S). Good clinical practice guidelines were strictly followed. This was designed as a pilot study, defined as cross-sectional cohort study, and STROBE guidelines were strictly followed. Upon written informed consent, 40 patients already included in a study at the Department of Neurology (ethics reference number # 2012-365-f-S) were recruited between March 2014 and February 2015 at the memory clinic. An evaluation followed at the Department of Periodontology, School of Dental Medicine, University of Münster, Germany. After neuropsychological testing and determination of total tau protein and Aß1-42 in CSF, the cohort included 20 patients with AD and 20 patients diagnosed with other forms of dementia (e.g. primary progressive aphasia or mild cognitive impairment) (noAD/DEM). AD and noAD/DEM diagnosis was made according to the 2011 guideline of the National Institute of Aging, Alzheimer’s Association workgroups (NIAA).^27^

All study participants were Caucasian, and all had a mini-mental status examination (MMSE) score ≥ 19.^12^ The age range of the patients was 30–65 years. They received routine neurological and neuro-psychological diagnostics due to their incipient cognitive/behavioural impairments and were subsequently examined dentally and periodontally. Serum and cerebrospinal fluid samples (CSF) were also taken.

The patients did not take any relevant medications, e.g. oral corticosteroids and/or cytostatic drugs > 20 mg/day, did not suffer from diabetes mellitus or anemia (Hb < 6 mmol/l), and were non-smokers or former smokers with more than 5 years of abstinence.

Patients were excluded from the study if 1) they did not read, sign or understand the informed consent form; 2) they had legal assistance due to their illness and its resulting neurocognitive/behavioural impairments; 3) they were not able to agree to participating in this study; 4) they were non-Caucasians, younger than 30 or older than 70 years; 5) they were current smokers; 6) they had taken antibiotics within the last 6 months; 7) they had a prophylaxis due to endocarditis or an antibiotic shield for artificial joints was necessary; 8) non-compliance with therapy or study protocol was expected; 9) female patients were pregnant or breast feeding.

### Clinical Assessment

After detailed information about the study protocol and procedure followed by signing the informed consent, all patients received an anamnestic interview. In case-report forms, data including age and gender, general medical conditions, diagnostic date of Alzheimer’s or other forms of dementia, and smoking habits were anonymously collected.

### Clinical Procedures – Neurological and Neuropsychological Examination

All patients were assessed using an extensive neuropsychological test battery covering all major neurocognitive domains, as described in detail elsewhere.^33^ Alongside specific tests for the domains, language, executive functions, practice and attention, assessments included the Consortium To Establish A Registry For Alzheimer’s Disease (CERAD) neuropsychological test battery.^1^ Neuropsychological testing, scoring and interpretation were done in accordance with the professional guidelines by a senior neuropsychologist (AJ). Further, patients and caregivers underwent comprehensive clinical interviews for medical and psychiatric history, a detailed neurological examination, structural magnetic resonance imaging (MRI) of the brain to evaluate focal atrophy patterns, and CSF analysis for dementia biomarker constellation (Aß1-42 and total tau). Final diagnoses of AD vs noAD/DEM were made in a multidisciplinary team consisting of senior neurologists, radiologists and clinical neuropsychologists according to current diagnostic criteria for AD.^27^

### Cerebrospinal Fluid (CSF) and Serum Sampling

Total amyloid beta (Aβ1-42) and total tau (t-tau) cerebrospinal fluid (CSF) levels were assessed by lumbar puncture. The kits “Innotest β-amyloid(1-42)” (Fujirebio, Hannover, Germany; Ref: 81576) and “hTau total ELISA” (Analytik Jena; Jena, Germany, Ref: 847-108000101) were used. The clinical cut-off value in the lab was 500 ng/l for both Aβ1-42 and t-tau. Aβ1-42 values below 500 ng/l and t-tau values above 500 ng/l were considered pathological.

### Clinical Procedures – Dental and Periodontal Examination

At first, digital orthopantograms were taken in all patients to assess the radiographic status of all teeth, including the average percentage of horizontal bone loss in relation to the cementoenamel junction, lesions with vertical bone loss, dental implants or endodontic restorations, and visible calculus. A calibrated periodontist (OL) recorded dental and periodontal parameters. In preparation for dental examination, teeth were dried using air and/or cotton rolls. For the diagnostic dental status, missing teeth, restorations, caries lesions, caries-free teeth and sufficient/insufficient restorations were determined using a mirror and a probe. The number of decayed, missing and filled teeth (DMFT) and the number of decayed, missing and filled surfaces (DMFS)^40^ were recorded.

Periodontal measurements were taken using a standardised manual periodontal probe with a tip diameter of 0.5 mm (UNC 15, Hu-Friedy; Chicago, IL, USA). Calibrations for the validation of intra-examiner reproducibility were performed on one subject not included in the study. During regular patient care, all clinical measurements were used to calibrate the examiner on two separate occasions on the same day, but at least 4 hours apart. Intraclass correlation analysis was used to calculate intra-examiner agreement for repeated measurements. The calibration was accepted if both measurements were similar in more than 90% (intraclass correlation coefficient >0.900).

Full-mouth periodontal charting including probing pocket depths (PPD), recessions as lack of gingival tissue (REC), and clinical attachment loss (CAL) described as loss of soft- (REC) and hard-tissue (PPD) integrity of each tooth was measured. Measurements were performed at six locations on each tooth (mesio-buccal, buccal, disto-buccal, mesio-oral, oral and disto-oral). Furcation involvement, described as bone loss between two or more roots of molars and premolars, was recorded (0: no bone loss in the furcation area, I: bone loss just above the furcation entrance; II: bone loss extent approximately one-third of the width of the tooth; III: continuous bone loss between roots).^14^

The full-mouth plaque score (FMPS) was recorded as the percentage of dental surfaces covered with plaque detected by the use of a periodontal probe. Full-mouth bleeding-on-probing (BOP) was assessed following probing pocket depth measurements based on the presence or absence of bleeding up to 30 s.^2,22,29,35^

All periodontal parameters were collected as data sheets using the standardised computer-tool Parostatus.de (Parostatus; Berlin, Germany); subgroups according to clinical attachment levels (CAL) and probing pocket depths (PPD) were automatically counted. Furthermore, epithelial and inflammatory areas as well as percentage of inflammation according to PISA were calculated.^28^

### Statistical Analysis

The primary endpoints of the study were differences in periodontal and general dental parameters in both groups. Following descriptive statistics of primary endpoints for both groups, analytical statistics were performed. First, both groups were tested for a normal distribution and subsequently compared using Student’s t-test, Wilcoxon matched-pairs signed rank-test or chi-squared test, and Fischer’s exact test using SPSS (SPSS IBM; Armonk, NY, USA). The significance level was set at p = 0.05.

## Results

### Demographic and Neurological Variables

In total, 40 patients were recruited and fulfilled the inclusion criteria. It included two groups of 20 patients each with AD and noAD/DEM. A low CSF level of β-amyloid1-42 (p = 0.004) and elevated t-tau in CSF (p = 0.006) in the AD vs noAD/DEM groups confirmed the diagnosis of AD in the respective group. The MMSE^12^ did not differ statistically significantly between the two groups (p = 0.389) (Table 1).

**Table 1:** Demographics and neurological variables as mean ± SD in patients diagnosed with Alzheimer’s disease (AD) and with other form of dementia (noAD/DEM)

	AD	noAD/DEM	p-value
Patients (n)/females/males	20/11/9	20/8/12	1.000
Age (years)	58.3 ± 5.2	61.1 ± 9.9	0.158
β-amyloid1-42 (ng/l CSF)	343 ± 82	827 ± 370	<0.004**
Total Tau (ng/l CSF)	841 ± 307	532 ± 365	0.006**
MMSE	22.1 ± 5.4	23.8 ± 5.4	0.389

**Statistical significance (p < 0.001); CSF= cerebrospinal fluid; MMSE = mini-mental state examination.

### Dental Parameters

All patients were fully or partially dentate; none were edentulous. The mean number of teeth was 25 (AD: 26.0±6.0; noAD/DEM: 24.0±8.0), with no statistically significant difference between groups (p = 0.395) (Table 2). On average, 18.0 teeth were restored. In the AD group, the mean DMFT was 17.2 ± 6.7 and mean DMFS was 61.8 ± 37.0. In the noAD/DEM group, the DMFT was 18.9 ± 5.5 and the DMFS was 72.4 ± 35.8 (Table 2).

**Table 2:** Dental parameters as mean ± SD of patients diagnosed with Alzheimer’s disease (AD) and with other form of dementia (noAD/DEM)

	AD	noAD/DEM	p-value
Teeth	26.0 ± 6.0	24.0 ± 8.0	0.395
Missing teeth	5.6 ± 4.1	7.6 ± 5.5	0.347
Oral malodor	2.0	5.0	0.220
Caries lesions	1.2 ± 1.3	0.4 ± 0.5	0.180
Fillings	6.0 ± 4.0	6.5 ± 3.3	0.559
Root-canal treatments	1.1 ± 1.3	1.2 ± 1.1	0.054
Patients with restored teeth	18.0	18.0	1,000
Crowns	2.45 ± 1.9	3.05 ± 2.6	0.762
Bridges	1.3 ± 1.2	1.1 ± 0.8	0.810
Implants	0.5 ± 0.8	0.4 ± 0.7	0.689
Age restorations	11.2 ± 6.7 years	9.1 ± 5.4 years	0.752
DMFT	17.2 ± 6.7	18.9 ± 5.5	0.497
DMFS	61.8 ± 37.0	72.4 ± 35.8	0.185

Statistical significance (p > 0.05); DMFS = decayed, missed, filled teeth; DMFS = decayed, missed, filled surfaces.

### Periodontal Parameters

**Fig 1:**
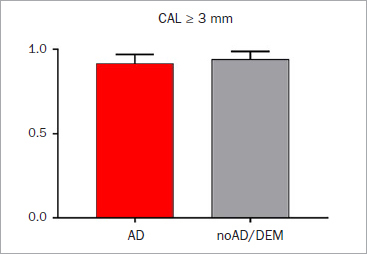
Clinical attachment loss (CAL) ≥ 3 mm in patients diagnosed with Alzheimer’s disease (AD) and with other form of dementia (noAD/DEM).

**Fig 2:**
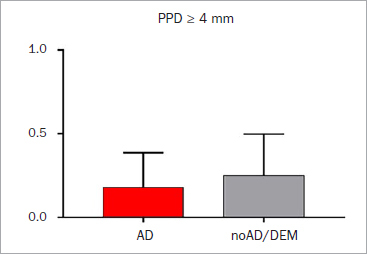
Probing pocket depth (PPD) ≥ 4 mm in patients diagnosed with Alzheimer’s disease (AD) and with other form of dementia (noAD/DEM).

Periodontal indices were also not statistically significantly different between the groups (Table 3). Around 85% of sites showed signs of periodontal destruction (clinical attachment loss ≥ 3 mm) (Fig 1). Additionally, the number of pockets with a PPD ≥4 mm did not vary statistically significant (Fig 2). According to PISA,^28^ the whole epithelial area and the inflammatory area in the Alzheimer’s group were 1624.4 ± 423.4 mm^2^ and 887.6 ± 542.3 mm^2^, respectively, whereas the noAD/DEM group showed values of 2116.2 ± 1141.3 mm^2^ and 875.4 ± 499.7 mm^2^, respectively. This yielded percent inflammed areas of 54.1 ± 30.1% for the AD group and 53.1 ± 27.1% for the noAD/DEM group (Table 3).

**Table 3:** Periodontal variables as mean% ± SD of patients diagnosed with Alzheimer’s disease (AD) and with other form of dementia (noAD/DEM)

	AD	noAD/DEM	p-value
Periodontal therapy Treated patients / years ago	8.0 / 1.7 ± 2.1	7.0 / 3.9 ± 4.1	0.750
Inflammation% (PISA)	54.1 ± 30.1%	53.1 ± 27.1%	0.914
Whole inflammatory area mm^2^ (PISA)	887.6 ± 542.3	875.4 ± 499.7	0.957
Whole epithelial area mm^2^ (PISA)	1624.4 ± 423.4	2116.2 ± 1141.3	0.978
Full-mouth plaque score	64.3 ± 23.8	67.8 ± 26.4%	0.667
Bleeding-on-probing	54.7 ± 31.1%	52.0 ± 23.7%	0.763
Max radiographic bone loss	42.5 ± 25.3%	40.9 ± 32.3%	0.633
Clinical attachment loss 3-4 mm ± SD	48.4 ± 17.7%	38.4 ± 17.3%	0.091
Clinical attachment loss >5 mm	33.6 ± 25.8%	48.2 ± 28.4%	0.102
Probing pocket depth 4–6 mm	16.7 ± 13.2%	26.2 ± 20.2%	0.147

Statistical significance (p > 0.05); PISA=Periodontal Inflamed Surface Area.

## Discussion

This pilot study found no difference between AD and noAD/DEM patients with regard to oral and periodontal parameters.

Very poor dental conditions are documented in all forms of dementia, but no single previous study has analysed differences between AD and noAD/DEM, according to the results of a recently published review.^24^ Other reviews reported that individuals with all kinds of dementia had statistically significantly fewer teeth (mean difference: -1.25; 95% CI: -0.832, -5.89; p < 0.0001; n = 8 studies), and a statistically significantly higher number of decayed, missing and filled teeth.^37^ In a systematic review,^43^ the association between oral health, mainly assessed by number of teeth, and cognitive status was reported. A recently published review discussed tooth loss and an association between periodontal disease and dementia.^37^ A number of other studies have reported a possible association between periodontal disease and all forms of dementia while focusing on the number of teeth, tooth loss or the DMFT index.^7,13,17^ However, neither in reviews nor in other studies was a distinction made between different kinds of dementia, and when reporting an association with periodontal disease, no consistent definitions for periodontal disease according to consensus criteria^4,6,30^ were used.

Regarding an association of periodontal inflammation with disease initiation and disease progression of AD as reflected in tau protein and β-amyloid plaques, the advantages of the following study are: 1. We used commonly accepted diagnostic criteria for periodontal disease on a complete dentition; and 2. neurological and neuropsychological aspects were also part of the present study to distinguish between different forms of dementia and particularly to define AD patients. Therefore, detailed neurological examination, structural magnetic resonance imaging (MRI) of the brain to evaluate focal atrophy patterns, and CSF analysis for dementia biomarker constellation (Aß1-42 and t-tau) was used. Final diagnoses of AD vs noAD/DEM were made according the 2011 guideline of the National Institute of Aging, Alzheimer’s Association workgroups (NIAA).^27^ A multidisciplinary team consisting of senior neurologists, radiologists and clinical neuropsychologists as per current diagnostic criteria for AD made the final diagnosis, distinguished AD and noAD/DEM groups,^27^ and pointed out clear diagnostic differences.

Since the aim of this study was to compare oral and periodontal health within groups of patients with neurocognitive impairments, here AD and noAD/DEM, the attempt was made to exclude extensive memorty loss as a confounder. Therefore. both AD and noAD/DEM patients had a “mini-mental status examination” (MMSE) score ≥ 19.^12^ All patients were non-smoking Caucasians who were relatively young, 30-65 years old, with a mean age of 58.3 ± 5.2 in the AD group and 61.1 ± 9.9 in the noAD/DEM. This minimized all cause-related confounding factors, since both smoking and age are considered common risk factors for all types of dementia^44^ and periodontitis.^10^ Therefore, smoking and older age (>70 years) were clear exclusion criteria.

Possible pathological mechanisms by which periodontitis may contribute to AD were postulated. First, bacteria associated with periodontitis may spread from the periodontal region to the bloodstream and thence into other organs. Second, microbial toxins and inflammatory mediators enter and damage the vascular system.^23^ Compared to cognitively healthy controls with periodontitis, some studies showed that TNF-alpha levels were significantly higher in both AD and noAD/DEM patients susceptible to periodontitis.^19,32^ The present study confirmed this, as all patients suffered from periodontal disease.

Implications of all forms of dementia for oral health must always be considered in diagnostics. Other authors have shown that patients suffering from dementia are less capable of performing sufficient oral hygiene measures.^8^ Many studies include patients in nursing homes and focus on a lack of knowledge by nursing staff.^38^ A recently published study of older patients found the oral hygiene status among care-dependent dementia patients to be inacceptable, although the patients received assistance in oral care.^31^ This finding was explained with the resistance of demented patients towards oral hygiene care.^31^ In the present study, none of the younger patients lived in nursing home facilities or needed caregivers.

## Conclusion

The present data suggest that patients both with AD and noAD/DEM need special dental care to improve periodontal and oral health. Consequently, the need for dental care, especially in nursing homes, should be emphasised.
